# Composition Analysis of III-Nitrides at the Nanometer Scale: Comparison of Energy Dispersive X-ray Spectroscopy and Atom Probe Tomography

**DOI:** 10.1186/s11671-016-1668-2

**Published:** 2016-10-18

**Authors:** Bastien Bonef, Miguel Lopez-Haro, Lynda Amichi, Mark Beeler, Adeline Grenier, Eric Robin, Pierre-Henri Jouneau, Nicolas Mollard, Isabelle Mouton, Benedikt Haas, Eva Monroy, Catherine Bougerol

**Affiliations:** 1 Université Grenoble Alpes, F-38000 Grenoble, France; 2 INAC, CEA-Grenoble, 17 av. des Martyrs, F-38000 Grenoble, France; 3 LETI, CEA-Grenoble, MINATEC Campus, 17 av. des Martyrs, F-38000 Grenoble, France; 4 Institut NEEL, CNRS, 24 av. des Martyrs, F-38000 Grenoble, France

**Keywords:** Energy dispersive X-ray spectroscopy, Atom probe tomography, Nanoscale composition analysis, Quantitative composition analysis, III-Nitride nanostructures

## Abstract

The enhancement of the performance of advanced nitride-based optoelectronic devices requires the fine tuning of their composition, which has to be determined with a high accuracy and at the nanometer scale. For that purpose, we have evaluated and compared energy dispersive X-ray spectroscopy (EDX) in a scanning transmission electron microscope (STEM) and atom probe tomography (APT) in terms of composition analysis of AlGaN/GaN multilayers. Both techniques give comparable results with a composition accuracy better than 0.6% even for layers as thin as 3 nm. In case of EDX, we show the relevance of correcting the X-ray absorption by simultaneous determination of the mass thickness and chemical composition at each point of the analysis. Limitations of both techniques are discussed when applied to specimens with different geometries or compositions.

## Background

As optoelectronic devices push towards higher performance, engineering the quantum confinement at the nanoscale become the key for device design. However, such a fine tuning of the physical properties faces one major bottleneck, namely the accurate determination of alloy compositions at the nanometer scale. For ternary or quaternary alloys, methods based on the analysis of the lattice parameters, such as X-ray diffraction, nanobeam electron diffraction [1], or geometrical phase analysis of high-resolution transmission electron microscopy images [2], cannot discriminate between strain and composition effects. Chemical analysis techniques such as energy dispersive X-ray spectroscopy (EDX) or atom probe tomography (APT) are better adapted to address this problem. However, their accuracy and spatial resolution need to be assessed in the case of III-nitrides. For instance, APT has been used to study in clustering in InGaN alloys [3–7] or AlN interlayers in AlGaN/AlN/GaN heterostructures [8]. However, getting quantitative composition information of nanometer-size nitride heterostructures from APT remains a challenge that requires careful optimization of the experimental conditions (temperature, laser wavelength and power, evaporation field) which are known to influence the ratio of the detected atomic species [9–11]. Combining data obtained by complementary approaches is a way to overcome these difficulties. Very recently, Griffiths et al. have reported the composition analysis of InGaN quantum wells from APT and quantitative scanning transmission electron microscopy (QSTEM) [12] which is an alternative approach to the TEM-based method first proposed by Rosenauer et al. [13]. Combining two chemical analysis techniques, namely EDX and APT, appears as an interesting alternative, the main problem to get accurate EDX quantitative data being linked to the absorption of the X-ray emission, which depends on the energy of the considered X-ray line, and the mass thickness of the sample.

## Methods

**Fig. 1:**
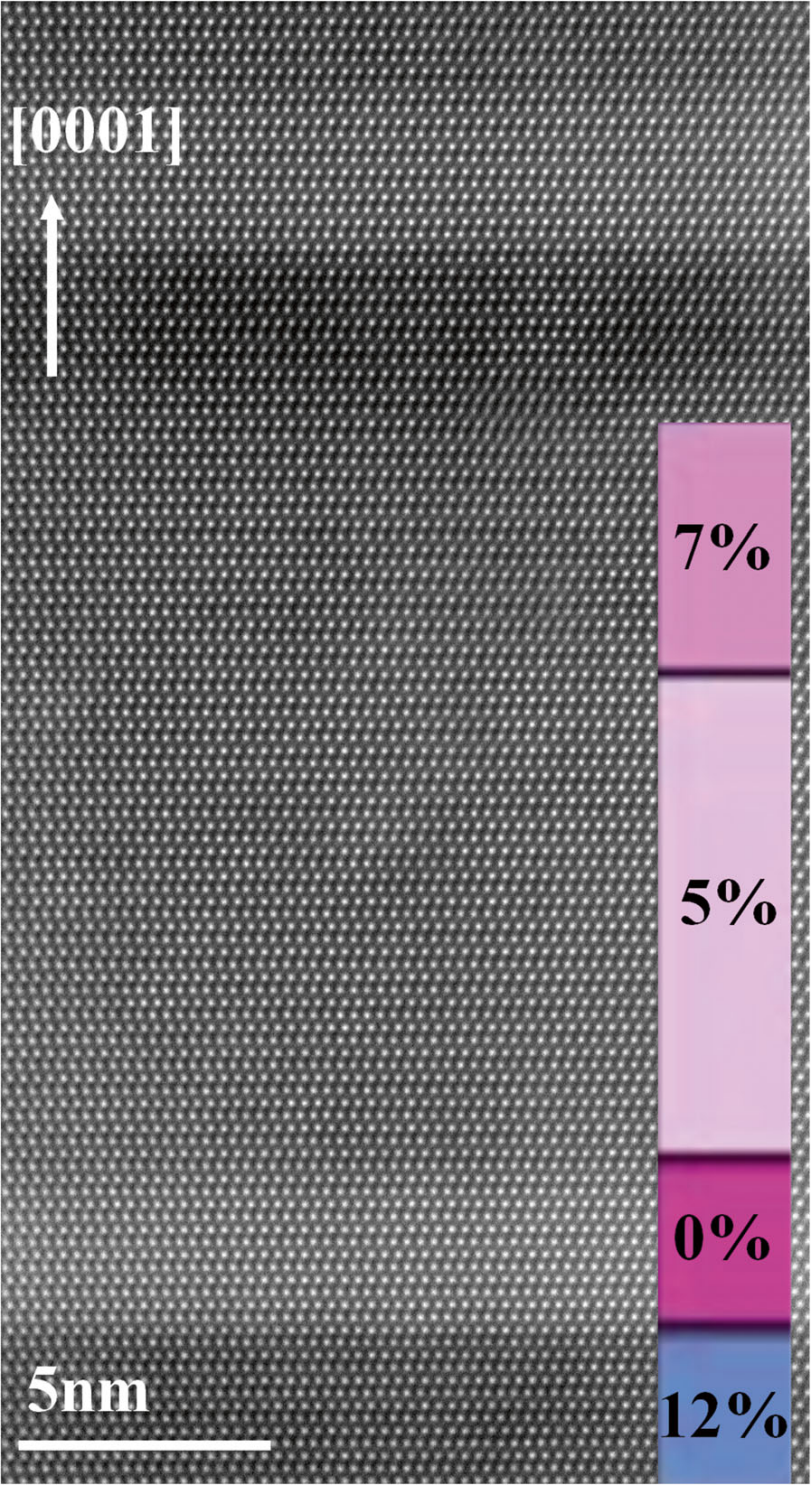
HAADF-STEM image taken along the [11-20] zone axis showing one period of the multilayer stack with a scheme of the stack given in the *inset*

The goal of this work was to evaluate and compare EDX and APT analysis of III-nitride alloys, in terms of spatial resolution and composition accuracy. For that purpose, we have chosen to investigate an AlGaN/GaN multilayer stack grown by plasma-assisted molecular beam epitaxy (PAMBE) consisting of a repetition (40 periods) of four AlGaN layers with nominal Al concentrations of 12, 0, 5, and 7% and nominal thicknesses of 3, 3, 11, and 6 nm, respectively. A high-angle annular dark-field (HAADF) scanning transmission electron microscopy (STEM) image of a period of the structure is shown in Fig. 1. The stack was deposited on a 30-nm-wide Al_0.05_Ga_0.95_N layer on a GaN-on-Si (111) template. Growth details and optical characterization can be found in ref. [14]. We will show that, after optimization of the acquisition and analysis parameters, both EDX and APT give comparable results with a composition accuracy better than ±0.6% even for layers as thin as 3 nm. The combination of both techniques paves the way to precise quantitative analysis of three-dimensional (3D) features at the nanoscale in nitride semiconductors.

## Results and Discussion

### Energy Dispersive X-ray Spectroscopy (EDX)

EDX data have been collected on an FEI-Osiris microscope operated at 200 kV and equipped with a Brücker EDX system consisting of four silicon drift detectors which ensure a high signal-to-noise ratio even at a probe size <1 nm. The sample has been prepared as wedge-shaped by focused ion beam (FIB) to study the influence of the thickness on the composition measurements. The quantitative EDX analysis has been carried out by two methods, namely the Cliff-Lorimer method (or *k*-factor method) [15] implemented in commercial EDX software and the *ζ*-factor method, first proposed by Watanabe et al. [16]. The Cliff-Lorimer method relates the concentration ratio of two elements to the ratio of the intensity peaks in the spectrum through the so-called *k*-factors. Its most serious limitation is the difficulty to correct the X-ray absorption, since it requires prior knowledge of the specimen thickness and density at the point of analysis, which are usually unknown. Such a correction is crucial for precisely quantifying light atoms like N and Al in AlGaN. On the other hand, the *ζ*-factor method takes into account X-ray absorption, thanks to the simultaneous determination of the mass thickness and composition of the sample at each point of analysis. Basically, the method uses an iterative procedure to compute the mass thickness, composition, and absorption correction terms. More details for thin lamella analysis can be found in the original paper of Watanabe et al. [16]. Although this new quantification procedure is still unavailable on commercial systems, our group is developing this new method using reference samples of known thickness and composition [17]. In the present case, the *ζ*-factors for N, Al, and Ga K-lines as well as Ga L-lines have been directly measured on the same equipment, at the same operating conditions, using the following as reference samples: GaN, Si_3_N_4_, and AlN for N; Al_2_O_3_, MgAl_2_O_4_, albite, orthoclase, and AlN for Al; and GaP, GaN, and GaAs for Ga.

**Fig. 2:**
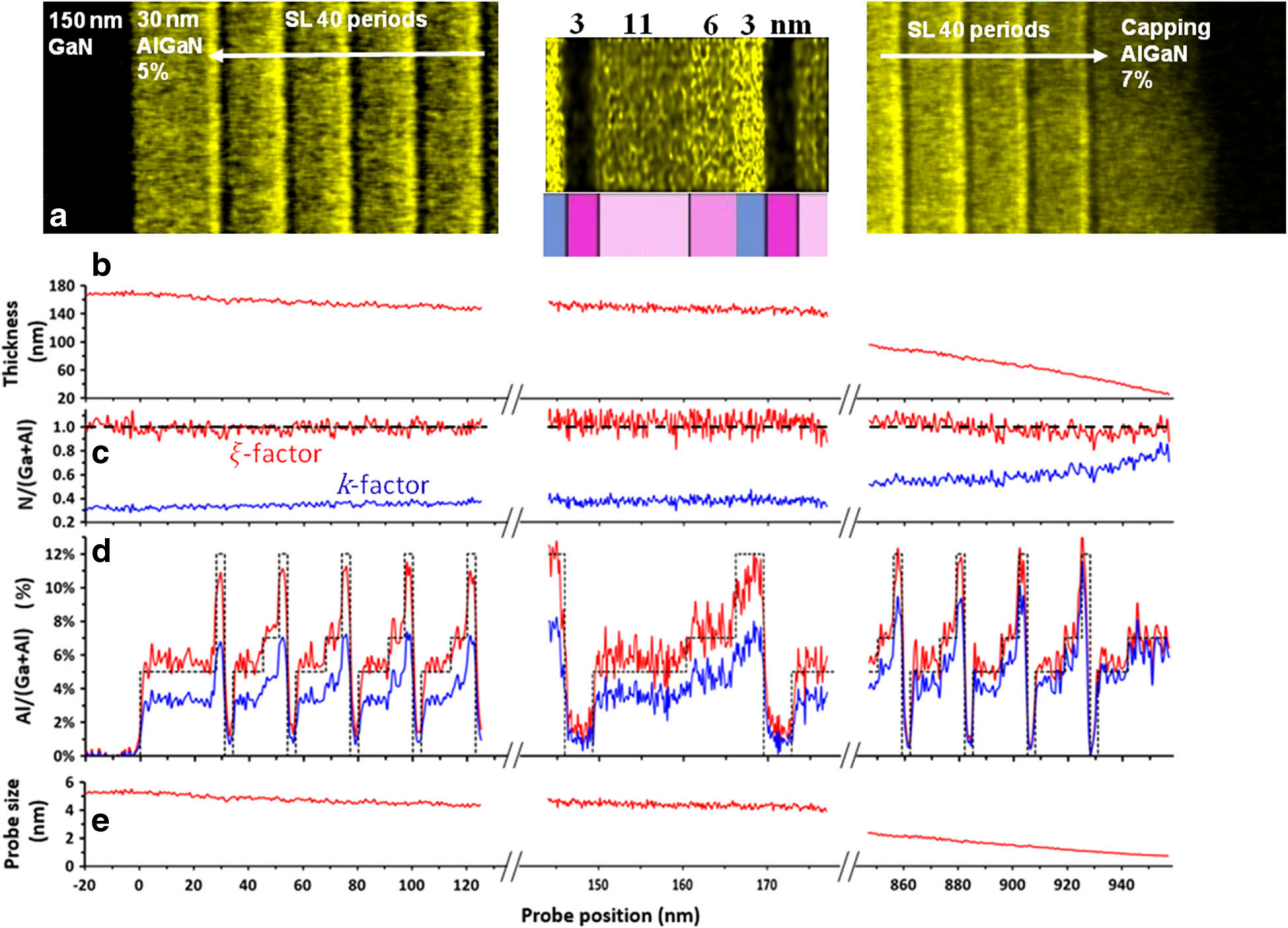
EDX analysis at different positions along the growth axis: close to the GaN buffer (*left*), zoom on four periods at an intermediate position (*middle*), and at the top part (*right*). **a** Al chemical maps. **b** Specimen thickness. **c** N/(Ga+Al) ratio. **d** Al/(Ga+A) ratio. **e** Probe size

Figure 2a shows Al chemical maps recorded at different positions along the growth axis: close to the GaN buffer layer (left), an enlargement of a period at an intermediate depth (middle), and at the top part (right). The variation of the sample thickness over these different regions, retrieved from the *ζ*-factor analysis, is displayed in Fig. 2b. The variation of the N/(Ga+Al) ratio integrated along the height of Fig. 2a images is presented in Fig. 2c. In the case of the *ζ*-factor method (red curve), thanks to the point-by-point absorption correction, the N/(Ga+Al) ratio remains equal to one, as expected from the (Al,Ga)N stoichiometry. This is markedly different from the *k*-factor results (blue curve), for which the ratio is far too low (around 0.3) except for top of the sample (on the right) where the specimen thickness is below 100 nm. In this area, as the specimen thickness decreases, the measured N/(Ga+Al) ratio increases up to 0.8, but it never reaches 1.

Concerning the Al concentration, Fig. 2d shows the variation of the Al/(Ga+Al) ratio calculated using the *ζ*-factor (red curve) and *k*-factor (blue curve) methods. The spatial integration procedure was as described for the N/(Ga+Al) ratio, and the dotted line marks the ratio calculated from the nominal composition of each layer. All along the specimen, good agreement exists between the nominal (dotted) and *ζ*-factor curves (red), and the four different layers integrating the stack period can be clearly identified. The Al concentrations extracted from these measurements are 11.9, 0.4, 5.6, and 7.3%, to be compared to the 12, 0, 5, and 7% nominal values. The agreement is not so good for the *k*-factor results (blue). Moreover, in this case, the Al concentration decreases as the thickness of the specimen increases. This behavior outlines the relevance of the absorption correction, and thus the benefit of the *ζ*-factor method for light element analysis. The correction is even more crucial for thick specimens (>100 nm).

If we focus on the *ζ*-factor results, the composition measurement of the thinnest GaN layer (3 nm) gives an idea of the spatial resolution limit due to the probe broadening effect. A value of 1% is obtained in the thicker area of the specimen, whereas it was 0.4% in the thinnest region (note that 0% of Al is indeed measured in the GaN 150-nm-wide GaN buffer layer). Figure 2e depicts an estimation of the probe size, which is 1–2 nm in the thin region of the specimen (graphs on the right side), but reaches 5 nm as the specimen gets thicker (graphs on the left side). Consequently, the measurement of the 3-nm-wide layers is affected by the chemical composition of the adjacent layers, leading to an artificial decrease of the Al content in the 12% layer and the apparent presence of Al in the pure GaN layer. In turn, the concentration obtained in wider layers is reliable and accurate all along the specimen.

### Atom Probe Tomography (APT)

The same AlGaN sample has been studied by atom probe tomography (APT) using a CAMECA FlexTAP equipment with an ultraviolet laser (*λ* = 343 nm) operated in pulsed mode and at 20 K to improve both the spatial resolution and the quantification [18]. A FEI Strata 400S FIB system was employed to needle shape the sample [19]. A backside lift-out was used to reverse the analysis direction in order to evaporate first the 30-nm-wide Al_0.05_Ga_0.95_N layer grown below the multilayer stack. The evaporation of such a layer of known composition (Al/(Ga+Al) = 5.6% as determined from EDX) allows fine tuning of the analysis parameters (the laser energy is varied to obtain the correct Al/(Al+Ga) ratio) before reaching the stack. In our experiment, the best conditions corresponded to a laser energy of 1 nJ, and we measured a charge state ratio Ga++/Ga+ around 0.15 [11]. The atom positions and their time of flight were recorded, and the 3D sample volume was reconstructed using the IVAS software, which enables an iterative process in which the initial geometrical characteristics of the tip as well as the detector efficiency are varied until the thicknesses of the reconstructed layers correspond to the experimental values determined from STEM images with straight interfaces.

**Fig. 3:**
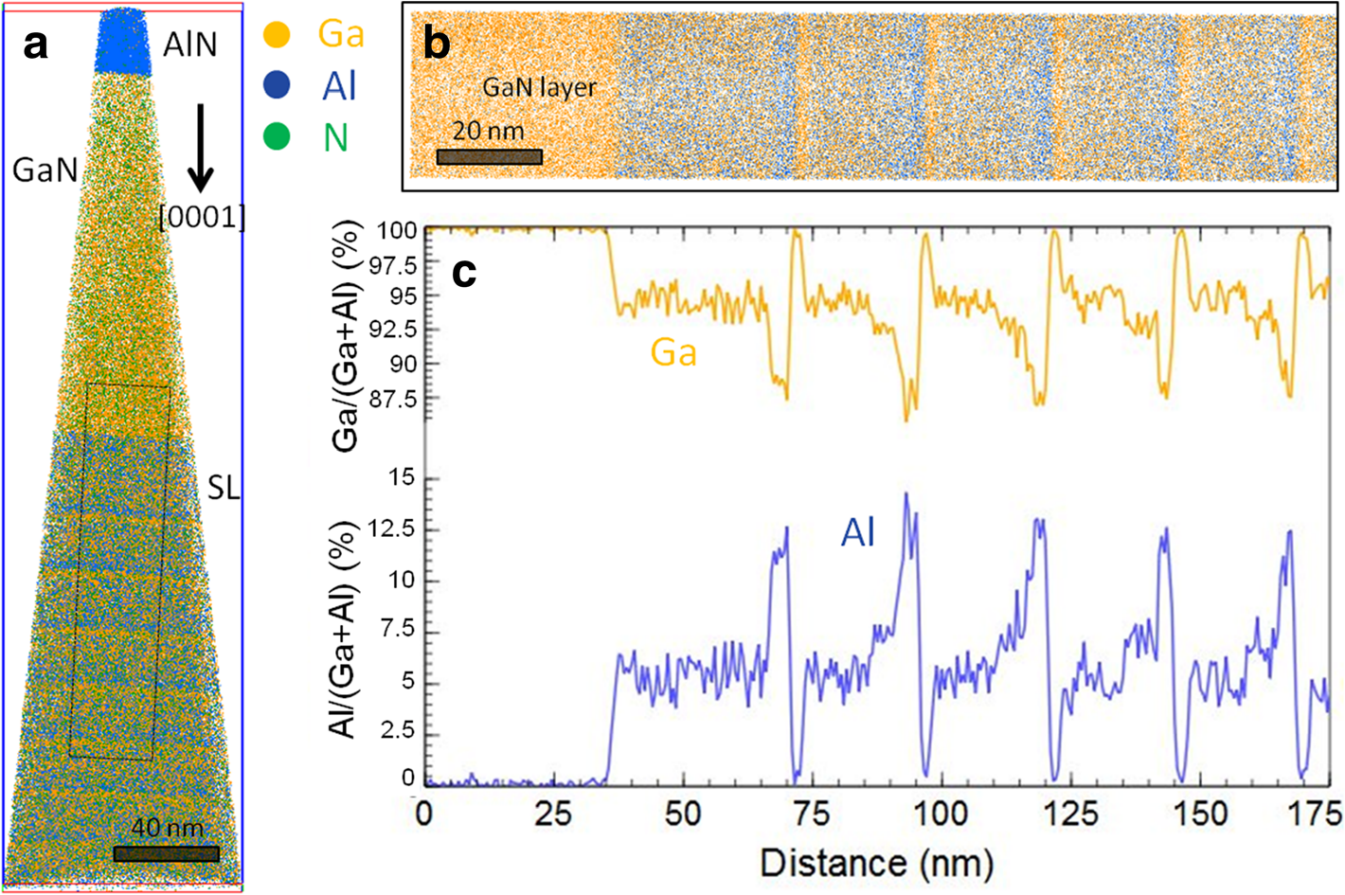
APT analysis. **a** 3D reconstruction of the sample tip evaporated including seven periods of the stack. **b** Zoom over the *black rectangle* marked in **a**. **c** Variation along the growth axis of the Ga/(Ga+Al) and Al/(Ga+Al) ratios for the volume shown in **b**

A 3D reconstruction of the sample tip, starting from the Al_0.05_Ga_0.95_N buffer layer and including seven periods of the stack, is presented in Fig. 3a. A zoom corresponding to the region marked as a black rectangle is shown in Fig. 3b, whereas Fig. 3c reports the variation of the Ga/(Ga+Al) and Al/(Ga+Al) ratios along the growth axis. These ratios have been obtained by counting the number of Al and Ga atoms in sampling boxes of 17 × 17 × 0.5 nm^3^ with a moving step of 0.5 nm. The values of the Al/(Ga+Al) ratio for the four layers of a period are 12.6 ± 0.7, 0.6 ± 0.2, 5.4 ± 0.3, and 7.5 ± 0.4%, in good agreement with the values obtained from EDX. The slight deviation between the measured and nominal layer thicknesses can be associated to a decrease of the detection efficiency (40% instead of 62% expected for our APT equipment). This effect has been previously reported in the case of III-nitrides [9, 11] and indicates that some atoms are preferentially detected without correlation with the laser pulses or can be eventually evaporated as neutral species.

**Fig. 4:**
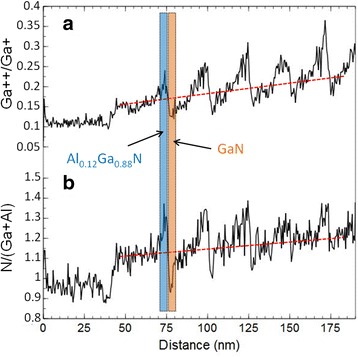
**a** Variation of the Ga++/Ga+ ratio during the evaporation of the volume shown in Fig. 3b. The trend of the average field is given by red dotted line. **b** Variation of the N/(Ga + Al) ratio

Concerning the spatial resolution, we notice that some Al (0.6 ± 0.2%) is detected in the 3-nm-wide GaN layers, whereas there are no Al counts emerging from the noise in the 150-nm-wide GaN layer. This is likely due to trajectory aberrations at the interfaces between GaN and adjacent AlGaN layers, linked to the difference in the evaporation fields of these layers. Figure 4a shows that the evolution of the Ga^++^/Ga^+^ ratio (indicating the evaporation field strength) during the tip evaporation presents marked discontinuities at the heterointerfaces. The red dotted line, which gives a trend of the average field, slowly increases during the evaporation and is associated to a slow increase of the N/(Al+Ga) ratio, as show in Fig. 4b. Increasing the Al composition of a layer increases the average III-N bond strength and therefore the field required for its evaporation. This field variation induces a variation in the N detection [19]. This implies that the composition analysis will bring reliable results only close to the region where the evaporation parameters have been optimized, i.e., a few periods in our case. This phenomenon will be further aggravated in case of layers having very different and higher Al content.

## Conclusions

This study shows that the correlative use of EDX in STEM and APT can provide reliable composition measurements in thin nitride layers, which are structural key parameters to understand electrical and optical properties of future devices. In particular, the Al content in AlGaN multilayers can be obtained in a reliable and comparable manner by both EDX and APT with a precision better than 0.6% even for layers of a few nanometers in width. It should be mentioned that the same sample has been investigated by X-ray diffraction ((Ө–2Ө) scans). The experimental diffractogram was perfectly fitted with the simulation performed considering the layers’ compositions given by EDX/APT and the layers’ thicknesses measured on STEM images, which confirms the reliability of our composition analyses. In case of EDX analysis, we have shown the necessity to use the *ζ*-factor approach which corrects precisely the X-ray absorption, thanks to the simultaneous determination of the mass thickness and chemical composition of the sample at each point of the analysis. This correction is particularly relevant for a reliable quantification of light atoms like N and Al, even for specimens thinner than 100 nm. The spatial resolution limit is imposed by the probe broadening effect, which is directly related to the specimen mass thickness and composition. This has to be taken into account especially when the width of the layers and the probe size are in the same range. Typically, the thinner the sample, the smaller the beam broadening; however, in very thin samples, the signal-to-noise ratio degrades, and hence the precision of the measurement. Regarding APT, the results are not affected by the specimen thickness, but precise quantification requires reference data provided by independent techniques, namely a layer of well-known composition to tune the laser energy at the first steps of the evaporation, and a STEM image of the sample to calibrate the 3D reconstruction. The challenge to quantify simultaneously Al, Ga, and N stands in the difference in the III-N bond strengths and consequently the field variations at the heterointerfaces.

## Abbreviations

3D: Three-dimensional
APT: Atom probe tomography
EDX: Energy dispersive X-ray spectroscopy
FIB: Focused ion beam
HAADF: High-angle annular dark-field
PAMBE: Plasma-assisted molecular beam epitaxy
QSTEM: Quantitative scanning transmission electron microscope
STEM: Scanning transmission electron microscopy
